# Flotillin: A Promising Biomarker for Alzheimer’s Disease

**DOI:** 10.3390/jpm10020020

**Published:** 2020-03-26

**Authors:** Efthalia Angelopoulou, Yam Nath Paudel, Mohd. Farooq Shaikh, Christina Piperi

**Affiliations:** 1 Department of Biological Chemistry, Medical School, National and Kapodistrian University of Athens, 11527 Athens, Greece; angelthal@med.uoa.gr; 2 Neuropharmacology Research Strength, Jeffrey Cheah School of Medicine and Health Sciences, Monash University Malaysia, Bandar Sunway 47500, Selangor, Malaysia; yam.paudel@monash.edu

**Keywords:** flotillin, Alzheimer’s disease, biomarker, exosomes, beta-amyloid, Tau

## Abstract

Alzheimer’s disease (AD) is characterized by the accumulation of beta amyloid (Aβ) in extracellular senile plaques and intracellular neurofibrillary tangles (NFTs) mainly consisting of tau protein. Although the exact etiology of the disease remains elusive, accumulating evidence highlights the key role of lipid rafts, as well as the endocytic pathways in amyloidogenic amyloid precursor protein (APP) processing and AD pathogenesis. The combination of reduced Aβ42 levels and increased phosphorylated tau protein levels in the cerebrospinal fluid (CSF) is the most well established biomarker, along with Pittsburgh compound B and positron emission tomography (PiB-PET) for amyloid imaging. However, their invasive nature, the cost, and their availability often limit their use. In this context, an easily detectable marker for AD diagnosis even at preclinical stages is highly needed. Flotillins, being hydrophobic proteins located in lipid rafts of intra- and extracellular vesicles, are mainly involved in signal transduction and membrane–protein interactions. Accumulating evidence highlights the emerging implication of flotillins in AD pathogenesis, by affecting APP endocytosis and processing, Ca^2+^ homeostasis, mitochondrial dysfunction, neuronal apoptosis, Aβ-induced neurotoxicity, and prion-like spreading of Aβ. Importantly, there is also clinical evidence supporting their potential use as biomarker candidates for AD, due to reduced serum and CSF levels that correlate with amyloid burden in AD patients compared with controls. This review focuses on the emerging preclinical and clinical evidence on the role of flotillins in AD pathogenesis, further addressing their potential usage as disease biomarkers.

## 1. Introduction

Alzheimer’s disease (AD) is pathologically characterized by the accumulation of beta amyloid (Aβ), a peptide of 40 or 42 amino acids, in extracellular senile plaques, as well as intracellular neurofibrillary tangles (NFTs) mainly consisting of tau protein [1,2]. Aβ is generated by the sequential cleavage of the transmembrane amyloid-β precursor protein (APP) via β- and γ-secretases [1]. It has been shown that amyloidogenic APP processing mainly occurs in lipid rafts, which are caveolae-like membrane microdomains enriched in sphingolipids, glycolipids, and cholesterol, serving as dynamic platforms for signal transduction, protein trafficking, and clathrin-independent endocytosis [3,4]. Most AD cases are sporadic, whereas a small percentage (1%–5%) of them are caused by mutations in genes including *APP*, *presenilin-1*, and *presenilin-2* [5]. Although the exact etiology and pathophysiological mechanisms of AD remain elusive, growing preclinical and clinical evidence highlights the key role of the homeostasis of lipid rafts and both intra- and extracellular vesicles in AD pathogenesis, even at early stages of the disease [6].

Currently, there is no easy and objective method available for early AD diagnosis. The combined estimation of reduced Aβ_42_ levels and increased phosphorylated tau protein levels in the cerebrospinal fluid (CSF) is the most well established biomarker for the diagnosis of the disease, along with Pittsburgh compound B and positron emission tomography (PiB-PET) for amyloid imaging [7,8,9]. Although both methods can relatively reliably detect AD at prosymptomatic stages [8,9], the lumbar puncture required for CSF collection is an invasive procedure demanding specialized staff technical skills and cannot be carried out in primary care settings [10]. On the other hand, PiB-PET is quite expensive and not available worldwide. Specific patterns of various sets of plasma proteins or phospholipids have been proposed as promising AD biomarkers by several studies, usually involving specialized experimental procedures, such as mass spectrometry, or necessitating the measurement of multiple factors [7]. In this context, an easily accessible and detectable marker with high sensitivity and specificity for AD diagnosis, reflecting Aβ burden or underlying AD pathophysiological mechanisms even at preclinical stages, is highly needed.

Flotillins are highly conserved hydrophobic myristoylated and palmitoylated proteins that belong to the prohibitin (PHB) family [3]. They are located in the inner part of the plasma membrane and play an essential role in the formation of lipid rafts [3,4]. Moreover, intracellular and extracellular vesicles are enriched in flotillins, whose subcellular localization is constantly changing [3,4]. Flotillins were first detected in the lung plasma membrane of mice, described as “float like a flotilla of ships in the Triton-insoluble, buoyant fraction” [3], and since then they have been widely utilized as markers of lipid rafts and exosomes for many years. In metazoans, there are two flotillin paralogues, termed as flotillin-1/reggie-2 and flotillin-2/reggie-1. Within cells, flotillins form flotillin-1/flotillin-2 hetero-oligomers mediated by C-terminal interactions [11]. Their expression is particularly high in cell types lacking caveolin, including neurons. Functionally, flotillins are implicated in various cellular processes, including membrane–cytoskeletal interaction, membrane and vesicular trafficking, signal transduction, axonal regeneration, cell migration, and clathrin-independent endocytosis. It has been demonstrated that flotillins (mainly flotillin-1) are abundantly expressed in pyramidal neurons in the cortex, as well as in the astrocytes of the white matter of normal human brain tissue [12]. Notably, their levels have been shown to be higher in brain samples from non-demented patients with amyloid plaques, subjects with Down syndrome (who overexpress APP), and AD patients compared with non-demented individuals without amyloid plaques [12], suggesting a key role in AD pathogenesis. Since then, several preclinical and human studies have investigated the implication of flotillins in AD pathophysiology, also highlighting their potential as promising molecular biomarkers.

**Figure 1:**
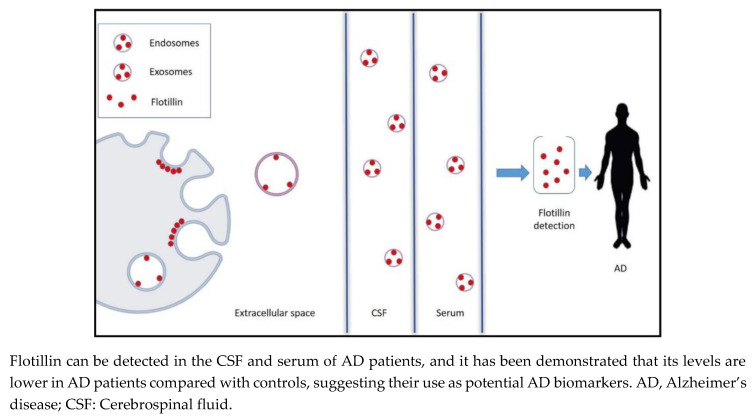
Lipid rafts, endosomes, and exosomes are enriched in flotillin.

In this mini review, we summarize the emerging preclinical and clinical evidence on the role of flotillins in AD pathogenesis, and discuss their potential usage as biomarker candidates (Figure 1) for AD along with possible limitations.

## 2. The Role of Endocytic Pathway and Exosome Release in AD Pathogenesis

Abnormal APP processing, trafficking, and turnover are considered to play a major role in AD pathogenesis [1]. Several studies indicate that amyloidogenic APP processing is predominantly carried out in lipid rafts, since APP, Aβ, presenilin-1, β-secretase, and specific components of the γ-secretase complex have been located in the membrane microdomains of neurons [13,14,15,16,17]. On the contrary, non-amyloidogenic APP processing usually occurs in areas of the plasma membrane that are enriched in phospholipids [18]. However, there is also evidence suggesting that amyloidogenic APP cleavage may occur outside lipid rafts. In particular, membrane cholesterol reduction in hippocampal neuronal cells from AD patients has been associated with enhanced β-site APP-cleaving enzyme 1 (BACE1)–APP colocalization and increased Aβ load, implying that amyloidogenic APP processing may be carried out in more fluid membrane domains [19]. In addition, depletion of the cholesterol-synthesizing enzyme seladin-1 in mice has been shown to be related to decreased cholesterol levels, disrupted cholesterol-rich detergent-resistant membrane domains (DRMs), displacement of BACE1 from DRMs to membrane fractions containing APP, enhanced APP cleavage, and increased Aβ load [20]. It has been proposed that BACE1 in cholesterol-rich membrane domains could represent a relatively inactive pool of the enzyme, which may be transferred to APP-containing domains under specific conditions. Based on these contradictory findings, more studies are needed in order to clarify this issue. Aβ can be produced by proteolytic cleavage of APP in the endoplasmic reticulum (ER) and *trans*-Golgi network, resulting in the formation of secretory vesicles. Alternatively, after APP internalization it can be directed from the plasma membrane into the endosomal system and subsequently targeted to lysosomes [21,22]. Early endosomes are the first vesicles of endocytic pathway responsible for APP endocytosis [23]. Internalization of APP into endosomes has been required for its cleavage by BACE1 [24], which proteolyzes APP [25]. Aβ generated in early endosomes has been also shown to be transported into late endosomes in a retrograde manner, leading to their fusion with cellular membrane and subsequent extracellular secretion as exosomes [26]. Impaired APP endocytosis has been associated with reduction in the secretion of Aβ in vitro [27], while abnormally enlarged endosomes have been identified in early stages of sporadic AD [23]. Therefore, dysregulation of the endocytic pathway has been implicated in AD pathophysiology, mainly by affecting APP processing and trafficking.

Exosomes are small membrane vesicles (20–120 nm in diameter) derived from endosomes through the formation of multivesicular bodies, being able to transport their internalized content into recipient cells, thus mainly contributing to cell-to-cell communication [28]. Exosomes can be secreted by various cell types, including neurons, astrocytes, oligodendrocytes, and microglia in the extracellular space and subsequently CSF, containing several kinds of cargos, including RNAs, micro-RNAs, and proteins [29,30]. It has been suggested that exosomes may be involved in AD pathogenesis by affecting Aβ metabolism and aggregation, as well as through tau-related molecular mechanisms [31]. More specifically, in vitro evidence has indicated that neuron-derived exosomes can bind to and promote conformational alterations in extracellular Aβ, leading to the formation of nontoxic amyloid fibrils. They can also enhance Aβ clearance by microglia through the facilitation of Aβ transportation to lysosomes for degradation [31]. Exosome secretion by neurons has been shown to be regulated by enzymes involved in the metabolism of sphingolipids, such as sphingomyelin synthase 2 (SMS2) and neutral sphingomyelinase 2 (nSMase2) [31]. Upregulation of exosome release with SMS2 siRNA use has been associated with increased Aβ uptake by microglia and reduced levels of extracellular Aβ in vitro [31]. In vivo evidence has revealed that exogenous intracerebral injection of neuroblastoma-derived exosomes into APP transgenic mice led to decreased Aβ depositions and Aβ-induced synaptotoxicity in their hippocampus [32]. In this study, glycosphingolipids (GSLs) enriched with glycans, which are essential components of exosome membrane, have exerted a contributing role in Aβ binding and internalization by the exosomes [32]. Notably, apart from Aβ, exosomes also contain β- and γ-secretase complexes, full-length APP, as well as APP C-terminal fragments (CTFs) in APP transgenic mice, highlighting their potential contribution to Aβ metabolism in the brain [33]. Apart from their role in Aβ pathology, secretion of phosphorylated tau proteins has been shown to be at least partially exosome-mediated in tauopathy models in vitro [34]. Moreover, exosome-associated phosphorylated tau has been found released in the cerebrospinal fluid of AD patients [34], further confirming the significant implication of exosomes in tau-related pathophysiology in human AD. It has been also demonstrated that exosomes can attenuate the Aβ-mediated disruption of synaptic plasticity in vivo [35]. Another study has shown that Aβ_1–42_ treatment inhibited exosome release from astrocytes, as demonstrated by decreased flotillin levels in vitro, by inducing c-Jun N-terminal kinase (JNK) signaling pathway [29]. Furthermore, exosomes, as Aβ carriers, have been proposed to contribute to the prion-like spreading of Aβ aggregates in AD [5]. Collectively, these findings reveal the key role of exosomes in AD pathogenesis by affecting Aβ pathology, tau-related mechanisms, and Aβ-mediated neurotoxicity.

## 3. The Role of Flotillin in AD Pathogenesis

### 3.1. Evidence from Human Studies

Several human studies support the potential implication of flotillin in AD pathogenesis. In this regard, it has been indicated that flotillin accumulates in the endosomes of neurons of AD patients’ brains [36], possibly playing a key role in neuronal endosomal pathway. More specifically, flotillin-1 immunoreactivity has been shown to be higher in neurons bearing NFTs in the amygdala, hippocampus, and isocortex of AD patients compared with controls [36], and flotillin-1 was found to be co-localized with cathepsin-D, a lysosomal protease, indicating its contribution to lysosomal degradation [36]. Of note, some neurons bearing NFTs did not contain flotillin-1, implying a possible secondary reduction in synthesis of flotillin-1 in these cells [36]. Flotillin-1 has been also demonstrated to be highly enriched in extracellular Aβ plaques and neurons bearing NFTs in brain specimens of AD patients compared with PD patients and controls [4]. Furthermore, it has been reported that flotillin-positive extracellular vesicles (EVs) isolated from the CSF and plasma of sporadic, late-onset AD patients contained an increased amounts of Aβ42 in their external surface compared with age-matched neurologically healthy controls, which could be internalized by cortical neurons and acted in a neurotoxic manner, by impairing Ca^2+^ homeostasis, causing mitochondrial dysfunction, increasing the vulnerability of neurons to excitotoxicity, and triggering neuronal apoptosis [37]. On the contrary, the EVs derived from controls displayed no neurotoxic effects [37]. These effects were also observed in vivo in transgenic APP and presenilin-1 mice, as well *as* in vitro in neural cells expressing presenilin-1 mutations of familial AD [37]. It was also reported that impaired autophagy could enhance the release of EVs [37]. Of note, these flotillin-positive EV-mediated effects have been shown to be prevented via Aβ antibody treatment, highlighting the essential role of Aβ in the EV-induced neurotoxicity [37]. Furthermore, another study has demonstrated that flotillin-1-positive exosomes isolated from postmortem brain sections of AD patients were highly enriched in toxic Aβ oligomers compared with healthy controls, which could be transferred into recipient’s cultured neurons [38]. Disruption of the production, release, or uptake of these exosomes was shown to attenuate the spreading of Aβ oligomers and Aβ-induced neurotoxicity [38]. Furthermore, a neuropathological study in brain tissues from AD patients has also reported that flotillins are also present in granulovacuolar degeneration (GVD) bodies [39], which are basophilic perinuclear vacuoles accumulating in neurons of AD patients [40]. Moreover, it has been indicated that levels of flotillin-2, along with other endocytic-associated proteins, were increased in brain sections of older non-demented humans [41]. Given the fact that age is a significant risk factor for AD development, these data further strengthen the potential contribution of flotillins in the pathophysiology of AD. These findings suggest that flotillin-positive exosomes may play a key role in AD pathogenesis and particularly, be implicated in the prion-like propagation of Aβ pathology in AD.

### 3.2. Evidence from In Vivo Models

In addition to human studies, a growing body of preclinical evidence demonstrates the key role of flotillin in the pathogenesis of AD, shedding more light into the underlying molecular mechanisms (Table 1).

More specifically, flotillin-1 was present in endosomes of neurons in the amygdala, hippocampus, and isocortex of transgenic mice expressing human mutant APP and presenilin-1 [21]. Furthermore, intracellular Aβ has been shown to accumulate in early and late endosomes of the endocytic pathway that is positive for flotillin-1 in transgenic ArcAβ mice [4], overexpressing human APP 695 (and containing the Swedish and Arctic mutations in a single construct). Another study demonstrated that knockout of *flotillin-1* gene (with or without additional knockout of *flotillin-2* gene) in transgenic mice overexpressing APP and mutant presenilin-1 was associated with reduced Aβ accumulation and plaque deposition [50]. However, no significant differences in APP clustering or endocytosis were observed in mouse embryonic fibroblasts with no flotillin-1 expression [50]. Another study has indicated that treadmill exercise could inhibit amyloidogenic APP cleavage and subsequent Aβ production in the brain of APP and presenilin-1 transgenic mouse models, by suppressing lipid raft formation and flotillin-1 levels [51]. Furthermore, it has been reported that treatment of senescence-accelerated mouse prone 8 mice with *Yizhijiannao* granule, a Chinese medicinal compound, could downregulate flotillin-1 levels in the temporal lobes of the animals [52]. Interestingly, it has been demonstrated that copper could inhibit the association of flotillin-2 with lipid rafts by redistributing it into non-raft fractions, thus decreasing the endocytosis and processing of APP in membrane microdomains both in vitro and in mutant APP transgenic mice [49]. Previous works have shown that copper can attenuate Aβ production [54], and suppression of flotillin-2-mediated APP processing in rafts may represent one potential underlying mechanism. On the other hand, there is also evidence indicating that clathrin-independent endocytic pathways may not play a significant role in APP processing, since flotillin levels have been shown to remain unchanged in mutant APP transgenic mice, compared with proteins associated with clathrin-dependent pathways [53]. Nevertheless, these findings highlight the important implication of flotillins in the pathogenesis of AD, however, further studies are needed for deeper understanding of the underlying molecular mechanisms.

### 3.3. Evidence from In Vitro Studies

There is also a growing amount of in vitro evidence investigating the role of flotillins at a molecular level. Specifically, flotillin-1 was shown to directly interact with the intracellular domain of APP (AICD), the C-terminus of APP consisting of 57–59 amino acids, while the residues 189–282 of flotillin-1 were found essential for this interaction. [42]. In this context, it has been demonstrated that the overexpression of FKBP12, a protein interacting with AICD, could trigger the amyloidogenic APP processing pathway in vitro, possibly by altering the affinity of AICD to flotillin-1 [43]. AICD has been demonstrated to play an important role in signal transduction via the interaction with specific PTB domain-containing proteins, including X11 and Fe65 [55]. Flotillin-1 has been also found to interact with leucine-rich glioma inactivated 3 (LGI3), a protein that is implicated in the internalization of APP in neurons [44]. More specifically, flotillin-1 was indicated to stabilize LGI3, and downregulation of the LGI3/Flo1 complex directly affected APP trafficking, by disrupting the formation of exosomes [44]. Another study has revealed that knockdown of flotillin-2 by small interfering RNA (siRNA) could impair APP endocytosis in primary cultures of hippocampal neuronal cells, resulting in reduced Aβ production in tissue cell cultures [45]. In addition, this study has demonstrated that flotillin-2 was able to act as a scaffold protein and enhance APP clustering at the surface of the plasma membrane, leading to APP endocytosis via a clathrin-dependent pathway [45]. Moreover, it has been indicated that flotillin-1 could bind to BACE1, whereas flotillin-1 overexpression was associated with increased recruitment of BACE1 into rafts and reduced activity of β-secretase in vitro [46]. Another study showed that flotillin-1 was able to directly interact with the dileucine motif in the cytoplasmic domain of BACE1, thus affecting its endosomal sorting. The flotillin-2–BACE1 interaction was shown to be indirect at least partially via flotillin-1 [47]. Depletion of both flotillins was also indicated to be associated with more prominent perinuclear localization and accumulation of overexpressed BACE1 into late endosomes in HeLa cells in which the expression of flotillin expression was knocked down via lentiviral shRNAs, suggesting that flotillins may affect its subcellular localization [47]. Additionally, flotillin-1 knockdown was demonstrated to result in overexpression of BACE1, and flotillin-2 knockdown led to increased amyloidogenic processing of endogenous APP in the same cellular system, possibly by affecting BACE1 trafficking [47]. Therefore, flotillins play a major role in BACE1 trafficking and expression, thus affecting APP cleavage. Moreover, neuroblastoma cells that were transfected with mutant *dynamin (DNM) 2* gene, in which a specific polymorphism has been associated with late-onset AD in non-carriers of the apolipoprotein E-ε4 allele [56], displayed higher levels of flotillin, and APP was predominantly localized in the lipid rafts [48].

Taken together, flotillins have been shown to affect APP endocytosis and processing, Ca^2+^ homeostasis, mitochondrial dysfunction, neuronal apoptosis, Aβ-induced neurotoxicity, and prion-like spreading of Aβ, thus playing an emerging role in AD pathogenesis.

## 4. Flotillin as a Novel Biomarker Candidate for AD: Clinical Evidence

CSF analysis is considered as the most reliable biofluid for detection of biomarkers of the central nervous system (CNS) disorders since it allows a more accurate elucidation of the underlying molecular processes in the brain. However, its acquisition requires lumbar puncture, which is an invasive procedure. Alternatively, blood biomarkers present a more optimal approach, and enormous research efforts are made towards this direction. However, the abundance of proteins in the plasma compared with the CSF limits the efficacy of this strategy for several molecules including Aβ_42_, and the results of respective clinical studies are often inconsistent [57]. In this regard, plasma neuron-derived exosomes obtained from AD patients have been shown to contain higher levels of Aβ42 and phosphorylated tau protein compared with controls, highlighting their potential usage as AD biomarkers [57]. Several methodological concerns remain to be clarified for the estimation of exosomes per se in AD, since their detection in blood or CSF may be affected by conditions of sample storage, temperature, and the use of anticoagulants [57]. Although various techniques for the isolation of exosomes from biofluids have been developed, there is still no established validated reference method for their effective purification [57].

Flotillin, as one of the main components of exosomes, has been recently proposed as an alternative single molecule that could be used as an AD biomarker. Proteomic analyses have already indicated that flotillins are constituents of CSF-derived exosomes [58,59]. Importantly, flotillin levels can be relatively easily measured by ELISA method and/or immunoblot analysis [10], providing a significant advantage compared with other biomarker candidates that may require more expensive and specialized equipment.

Interestingly, a recent clinical study has indicated that flotillin levels were lower not only in the CSF, but also in the serum of AD patients, compared with subjects with mild cognitive impairment (MCI) or age-matched non-AD controls [10]. Additionally, CSF and serum flotillin levels have been shown to be reduced in patients with AD-related MCI, compared with non-AD-related MCI (determined by PiB-PET) [10]. Investigation of postmortem brain tissues further revealed that flotillin levels were also lower in the cerebroventricular fluid (CVF) samples obtained from AD patients compared with subjects with vascular dementia [10]. Furthermore, flotillin levels have been found to be negatively correlated with amyloid burden, as shown by the mean cortical PiB retention levels in PiB-PET [10]. However, flotillin-2 levels were shown elevated in brain sections of older non-demented humans compared with younger individuals [41], as well as levels of plasma neuron-derived exosomes were lower in aged HIV-infected individuals compared with controls [60], raising concerns regarding the efficacy of flotillin levels to effectively discriminate between AD and normal ageing. In this regard, flotillin levels were found to remain stable with advancing age in healthy controls in the abovementioned study [10], highlighting the possibility that their levels may not be affected by ageing process itself.

Although clinical evidence on the diagnostic utility of flotillins in AD is limited and the subject is still in its infancy, these findings strongly support the promising role of flotillin as a novel early CSF and serum diagnostic biomarker for AD (Figure 1). Future studies with larger sample size are therefore required for the validation of these results.

## 5. Discussion and Future Perspectives

Accumulating evidence highlights the emerging implication of flotillins in AD pathogenesis, through their implication in APP endocytosis and processing, Ca^2+^ homeostasis, mitochondrial dysfunction, neuronal apoptosis, Aβ-induced neurotoxicity, and prion-like spreading of Aβ. Importantly, there is also clinical evidence suggesting their potential use as biomarker candidates for AD, based on the reduced serum and CSF levels observed in AD patients compared with controls that correlated with amyloid burden.

On the other hand, there are specific challenges related to the development of flotillins as valid AD biomarkers. Firstly, it is important to note that flotillins are universally expressed by all cell types, and consist as a component of all subtypes of extracellular vesicles, independently to their density or size [61]. In addition, it has been indicated that the concentration of plasma neuron-derived exosomes was lower in neurocognitively impaired individuals (regardless of the cause) in comparison with controls [60]. Furthermore, flotillin-1 has been reported to be enriched in exosomes from CSF samples obtained from patients at early stage of severe traumatic brain injury, although no comparisons with control subjects were made in this study [62]. Moreover, levels of CSF exosomal α-synuclein have been shown to distinguish patients with Parkinson’s disease, dementia with Lewy bodies, and controls with other neurological disorders, and correlate well with cognitive impairment [63]. However, flotillin-1 levels were shown to be higher in neurons bearing NFTs in brain specimens of AD compared with PD patients [4]. Nevertheless, these findings raise important concerns about the specificity of flotillin detection in AD, and highlight the need for future comparative studies in age-matched patients with other neurological disorders.

Notably, accumulating evidence reveals the potential role of tissue flotillin levels as prognostic biomarkers in various types of solid tumors, including breast cancer and lung carcinoma [64]. Although data about plasma flotillin levels in cancer patients are lacking, other comorbidities including tumors should be also considered for the evaluation of flotillin levels in AD patients. Furthermore, preclinical evidence has demonstrated that several types of statins may alter the expression of flotillins in brain plasma membrane [65], implying that treatment with statins may affect flotillin levels in AD patients. In addition, the potential effects of anti-cholinesterase inhibitors, currently prescribed for AD patients, on flotillin levels should be also considered.

Another issue that should be mentioned is the selective detection of flotillin-1 and -2 levels in the CSF. In this context, it has been indicated that flotillin-2 levels were positively correlated with flotillin-1 levels in the CSF obtained from patients after severe traumatic brain injury [62]. Nevertheless, further research is required regarding the differential role of both types of flotillins in the serum and CSF of AD patients.

Apart from flotillins, another mechanism of clathrin-independent endocytosis involves caveolins, which have been also implicated in Aβ production [66]. Caveolin-1 levels have been increased in the cortex and hippocampus of brain specimens from AD patients compared with age-matched controls [67], indicating another potential endocytosis-associated biomarker candidate for AD that could be further investigated.

Importantly, it has been shown that specific gene polymorphisms of the *neuronal sortilin-related receptor* (*SORL1*), which is highly implicated in the APP endosomal trafficking, may affect the susceptibility to AD development [68]. It has been shown that specific polymorphisms of *flotillin-2* gene may be associated with coronary artery disease in the Chinese population. Given the connection of hypercholesterolemia with AD development [18,69], further research is needed for the potential link between these *flotillin-2* polymorphisms and AD risk.

## 6. Conclusions

Collectively, flotillin may serve as a single CSF or blood biomarker, or be used supplementary to CSF Aβ42 and tau levels, as well as PET neuroimaging findings for more efficient and earlier AD diagnosis. However, additional larger comparative studies with age-matched controls and patients with other neurodegenerative disorders are needed for the validation of their usage as biomarkers for AD in clinical settings.

## Figures and Tables

**Table 1:** In vitro and in vivo evidence about the role of flotillin in Alzheimer’s disease (AD).

S.N.	Type of Study	Main Findings	Reference
1	*In vitro*	Flotillin-1 directly interacted with the intracellular domain of APP (AICD), and the residues 189-282 of flotillin-1 were essential for this interaction	[42]
2	*In vitro*	FKBP12 overexpression enhanced the amyloidogenic APP processing, possibly by affecting the affinity of AICD to flotillin-1	[43]
3	*In vitro*	Flotillin-1 could stabilize LGI3, and LGI3/Flo1 complex downregulation could directly affect APP trafficking via the disruption of exosome formation	[44]
4	*In vitro*	si-RNA-mediated flotillin-2 knockdown impaired APP endocytosis in primary cultures of hippocampal neurons, and reduced Aβ production in tissue cell cultures Flotillin-2 acted as a scaffold protein and enhanced APP clustering at the plasma membrane and subsequent APP endocytosis in a clathrin-dependent manner	[45]
5	*In vitro*	Flotillin-1 bound to BACE1, whereas flotillin-1 overexpression was associated with increased recruitment into rafts and reduced activity of BACE1	[46]
6	*In vitro*	Flotillin-1 could directly interact with the dileucine motif in the cytoplasmic domain of BACE1, thus affecting its endosomal sorting Flotillin-2-BACE1 interaction was at least partially mediated by flotillin-1 Depletion of flotillin-1 and -2 led to increased accumulation of overexpressed BACE1 into late endosomes in shRNA-mediated flotillin knockdown HeLa cells Flotillin-1 knockdown resulted in overexpression of BACE1 Flotillin-2 knockdown increased amyloidogenic processing of APP	[47]
7	*In vitro*	Neuroblastoma cells transfected with mutant *DNM 2* gene displayed higher flotillin levels, and APP was mainly localized in the lipid rafts	[48]
8	*In vivo & in vitro*	Copper inhibited the association of flotillin-2 with lipid rafts via its redistribution into non-raft fractions, resulting in the reduction of endocytosis and processing of APP in membrane microdomains both in vitro and in mutant APP transgenic mice	[49]
9	*In vivo*	Endosomes of neurons in the amygdala, hippocampus, and isocortex of APP and presenilin-1 transgenic mice contained flotillin-1	[21]
10	*In vivo*	Intracellular Aβ was accumulated in early and late endosomes positive for flotillin-1 in transgenic ArcAβ mice	[4]
11	*In vivo*	Knockout of *flotillin-1* gene (with or without additional knockout of flotillin-2 gene) in APP and presenilin-1 transgenic mice was correlated with reduced Aβ accumulation and plaque formation No differences on APP clustering or endocytosis were indicated in mouse embryonic fibroblasts with flotillin-1 depletion	[50]
12	*In vivo*	Treadmill exercise inhibited amyloidogenic APP cleavage and Aβ production in APP and presenilin-1 transgenic mice by inhibiting flotillin-1 levels and lipid raft formation	[51]
13	*In vivo*	Treatment of senescence-accelerated mouse prone 8 mice with Yizhijiannao granule reduced flotillin-1 levels	[52]
14	*In vivo*	Flotillin levels were not altered in mutant APP transgenic mice, compared with proteins associated with clathrin-dependent pathways	[53]

AD, Alzheimer’s disease; APP, amyloid precursor protein; Aβ, amyloid β; LGI3, leucine-rich glioma inactivated 3; BACE1, beta-site APP cleaving enzyme 1; DNM, dynamin; FKBP12, FK506-binding protein; shRNAs, short hairpin RNAs; siRNAs, small interfering RNAs.

## Abbreviations

AD: Alzheimer’s disease; APP, Amyloid precursor protein; Aβ, Amyloid β; NFTs, neurofibrillary tangles;LGI3, Leucine-rich glioma inactivated 3; BACE1, Beta-site APP cleaving enzyme 1; DNM, Dynamin; FKBP12, FK506-binding protein; shRNAs, Short hairpin RNAs; siRNAs, Small-interfering RNAs; PiB-PET; Pittsburgh compound B and positron emission tomography; PHB, Prohibitin; ER, Endoplasmic reticulum;SMS2, Sphingomyelin synthase 2;GSLs, Glycosphingolipids; CTFs, C-terminal fragments; EVs, Extracellular vesicles; GVD, Granulovacuolar degeneration; CNS, Central nervous system; CVF, Cerebroventricular fluid; SORL1, sortilin-related receptor 1; JNK, C-jun N-terminal kinases.

## References

[1] Tiwari S., Atluri V., Kaushik A., Yndart A., Nair M. (2019). Alzheimer’s disease: Pathogenesis, diagnostics, and therapeutics. Int. J. Nanomed..

[2] Paudel Y.N., Angelopoulou E., Piperi C., Othman I., Aamir K., Shaikh M. (2020). Impact of HMGB1, RAGE, and TLR4 in Alzheimer’s Disease (AD): From Risk Factors to Therapeutic Targeting. Cells.

[3] Bickel P.E., Scherer P.E., Schnitzer J.E., Oh P., Lisanti M.P., Lodish H.F. (1997). Flotillin and epidermal surface antigen define a new family of caveolae-associated integral membrane proteins. J. Biol. Chem..

[4] Rajendran L., Knobloch M., Geiger K.D., Dienel S., Nitsch R., Simons K., Konietzko U. (2007). Increased Abeta production leads to intracellular accumulation of Abeta in flotillin-1-positive endosomes. Neurodegener. Dis..

[5] Xiao T., Zhang W., Jiao B., Pan C.Z., Liu X., Shen L. (2017). The role of exosomes in the pathogenesis of Alzheimer’ disease. Transl. Neurodegener..

[6] Tate B.A., Mathews P.M. (2006). Targeting the role of the endosome in the pathophysiology of Alzheimer’s disease: A strategy for treatment. Sci. Aging Knowl. Environ..

[7] Irizarry M.C. (2004). Biomarkers of Alzheimer disease in plasma. NeuroRx J. Am. Soc. Exp. Neurother..

[8] Racine A.M., Koscik R.L., Nicholas C.R., Clark L.R., Okonkwo O.C., Oh J.M., Hillmer A.T., Murali D., Barnhart T.E., Betthauser T.J. (2016). Cerebrospinal fluid ratios with Abeta42 predict preclinical brain beta-amyloid accumulation. Alzheimer’s Dement..

[9] Vlassenko A.G., Benzinger T.L., Morris J.C. (2012). PET amyloid-beta imaging in preclinical Alzheimer’s disease. Biochim. Biophys. Acta.

[10] Abdullah M., Kimura N., Akatsu H., Hashizume Y., Ferdous T., Tachita T., Iida S., Zou K., Matsubara E., Michikawa M. (2019). Flotillin is a Novel Diagnostic Blood Marker of Alzheimer’s Disease. J. Alzheimer’s Dis..

[11] Neumann-Giesen C., Falkenbach B., Beicht P., Claasen S., Luers G., Stuermer C.A., Herzog V., Tikkanen R. (2004). Membrane and raft association of reggie-1/flotillin-2: Role of myristoylation, palmitoylation and oligomerization and induction of filopodia by overexpression. Biochem. J..

[12] Kokubo H., Lemere C.A., Yamaguchi H. (2000). Localization of flotillins in human brain and their accumulation with the progression of Alzheimer’s disease pathology. Neurosci. Lett..

[13] Bouillot C., Prochiantz A., Rougon G., Allinquant B. (1996). Axonal amyloid precursor protein expressed by neurons in vitro is present in a membrane fraction with caveolae-like properties. J. Biol. Chem..

[14] Lee S.J., Liyanage U., Bickel P.E., Xia W., Lansbury P.T., Kosik K.S. (1998). A detergent-insoluble membrane compartment contains A beta in vivo. Nat. Med..

[15] Riddell D.R., Christie G., Hussain I., Dingwall C. (2001). Compartmentalization of beta-secretase (Asp2) into low-buoyant density, noncaveolar lipid rafts. Curr. Biol..

[16] Vetrivel K.S., Cheng H., Lin W., Sakurai T., Li T., Nukina N., Wong P.C., Xu H., Thinakaran G. (2004). Association of gamma-secretase with lipid rafts in post-Golgi and endosome membranes. J. Biol. Chem..

[17] Kokubo H., Kayed R., Glabe C.G., Yamaguchi H. (2005). Soluble Abeta oligomers ultrastructurally localize to cell processes and might be related to synaptic dysfunction in Alzheimer’s disease brain. Brain Res..

[18] Vetrivel K.S., Thinakaran G. (2010). Membrane rafts in Alzheimer’s disease beta-amyloid production. Biochim. Biophys. Acta.

[19] Abad-Rodriguez J., Ledesma M.D., Craessaerts K., Perga S., Medina M., Delacourte A., Dingwall C., De Strooper B., Dotti C.G. (2004). Neuronal membrane cholesterol loss enhances amyloid peptide generation. J. Cell Biol..

[20] Crameri A., Biondi E., Kuehnle K., Lutjohann D., Thelen K.M., Perga S., Dotti C.G., Nitsch R.M., Ledesma M.D., Mohajeri M.H. (2006). The role of seladin-1/DHCR24 in cholesterol biosynthesis, APP processing and Abeta generation in vivo. EMBO J..

[21] Langui D., Girardot N., El Hachimi K.H., Allinquant B., Blanchard V., Pradier L., Duyckaerts C. (2004). Subcellular topography of neuronal Abeta peptide in APPxPS1 transgenic mice. Am. J. Pathol..

[22] Yamazaki T., Koo E.H., Selkoe D.J. (1996). Trafficking of cell-surface amyloid beta-protein precursor. II. Endocytosis, recycling and lysosomal targeting detected by immunolocalization. J. Cell Sci..

[23] Cataldo A.M., Barnett J.L., Pieroni C., Nixon R.A. (1997). Increased neuronal endocytosis and protease delivery to early endosomes in sporadic Alzheimer’s disease: Neuropathologic evidence for a mechanism of increased beta-amyloidogenesis. J. Neurosci. Off. J. Soc. Neurosci..

[24] Vetrivel K.S., Thinakaran G. (2006). Amyloidogenic processing of beta-amyloid precursor protein in intracellular compartments. Neurology.

[25] Refolo L.M., Sambamurti K., Efthimiopoulos S., Pappolla M.A., Robakis N.K. (1995). Evidence that secretase cleavage of cell surface Alzheimer amyloid precursor occurs after normal endocytic internalization. J. Neurosci. Res..

[26] Rajendran L., Honsho M., Zahn T.R., Keller P., Geiger K.D., Verkade P., Simons K. (2006). Alzheimer’s disease beta-amyloid peptides are released in association with exosomes. Proc. Natl. Acad. Sci. USA.

[27] Perez R.G., Soriano S., Hayes J.D., Ostaszewski B., Xia W., Selkoe D.J., Chen X., Stokin G.B., Koo E.H. (1999). Mutagenesis identifies new signals for beta-amyloid precursor protein endocytosis, turnover, and the generation of secreted fragments, including Abeta42. J. Biol. Chem..

[28] Elkin S.R., Lakoduk A.M., Schmid S.L. (2016). Endocytic pathways and endosomal trafficking: A primer. Wien. Med. Wochenschr..

[29] Abdullah M., Takase H., Nunome M., Enomoto H., Ito J., Gong J.S., Michikawa M. (2016). Amyloid-beta Reduces Exosome Release from Astrocytes by Enhancing JNK Phosphorylation. J. Alzheimer’s Dis..

[30] Paolicelli R.C., Bergamini G., Rajendran L. (2019). Cell-to-cell Communication by Extracellular Vesicles: Focus on Microglia. Neuroscience.

[31] Yuyama K., Sun H., Mitsutake S., Igarashi Y. (2012). Sphingolipid-modulated exosome secretion promotes clearance of amyloid-beta by microglia. J. Biol. Chem..

[32] Yuyama K., Sun H., Sakai S., Mitsutake S., Okada M., Tahara H., Furukawa J., Fujitani N., Shinohara Y., Igarashi Y. (2014). Decreased amyloid-beta pathologies by intracerebral loading of glycosphingolipid-enriched exosomes in Alzheimer model mice. J. Biol. Chem..

[33] Perez-Gonzalez R., Gauthier S.A., Kumar A., Levy E. (2012). The exosome secretory pathway transports amyloid precursor protein carboxyl-terminal fragments from the cell into the brain extracellular space. J. Biol. Chem..

[34] Saman S., Kim W., Raya M., Visnick Y., Miro S., Saman S., Jackson B., McKee A.C., Alvarez V.E., Lee N.C. (2012). Exosome-associated tau is secreted in tauopathy models and is selectively phosphorylated in cerebrospinal fluid in early Alzheimer disease. J. Biol. Chem..

[35] An K., Klyubin I., Kim Y., Jung J.H., Mably A.J., O’Dowd S.T., Lynch T., Kanmert D., Lemere C.A., Finan G.M. (2013). Exosomes neutralize synaptic-plasticity-disrupting activity of Abeta assemblies in vivo. Mol. Brain.

[36] Girardot N., Allinquant B., Langui D., Laquerriere A., Dubois B., Hauw J.J., Duyckaerts C. (2003). Accumulation of flotillin-1 in tangle-bearing neurones of Alzheimer’s disease. Neuropathol. Appl. Neurobiol..

[37] Eitan E., Hutchison E.R., Marosi K., Comotto J., Mustapic M., Nigam S.M., Suire C., Maharana C., Jicha G.A., Liu D. (2016). Extracellular Vesicle-Associated Abeta Mediates Trans-Neuronal Bioenergetic and Ca(2+)-Handling Deficits in Alzheimer’s Disease Models. NPJ Aging Mech. Dis..

[38] Sardar Sinha M., Ansell-Schultz A., Civitelli L., Hildesjo C., Larsson M., Lannfelt L., Ingelsson M., Hallbeck M. (2018). Alzheimer’s disease pathology propagation by exosomes containing toxic amyloid-beta oligomers. Acta Neuropathol..

[39] Nishikawa T., Takahashi T., Nakamori M., Yamazaki Y., Kurashige T., Nagano Y., Nishida Y., Izumi Y., Matsumoto M. (2014). Phosphatidylinositol-4,5-bisphosphate is enriched in granulovacuolar degeneration bodies and neurofibrillary tangles. Neuropathol. Appl. Neurobiol..

[40] Woodard J.S. (1962). Clinicopathologic significance of granulovacuolar degeneration in Alzheimer’s disease. J. Neuropathol. Exp. Neurol..

[41] Alsaqati M., Thomas R.S., Kidd E.J. (2018). Proteins Involved in Endocytosis Are Upregulated by Ageing in the Normal Human Brain: Implications for the Development of Alzheimer’s Disease. J. Gerontol. Ser. A Biol. Sci. Med Sci..

[42] Chen T.Y., Liu P.H., Ruan C.T., Chiu L., Kung F.L. (2006). The intracellular domain of amyloid precursor protein interacts with flotillin-1, a lipid raft protein. Biochem. Biophys. Res. Commun..

[43] Liu F.L., Liu T.Y., Kung F.L. (2014). FKBP12 regulates the localization and processing of amyloid precursor protein in human cell lines. J. Biosci..

[44] Okabayashi S., Kimura N. (2010). LGI3 interacts with flotillin-1 to mediate APP trafficking and exosome formation. Neuroreport.

[45] Schneider A., Rajendran L., Honsho M., Gralle M., Donnert G., Wouters F., Hell S.W., Simons M. (2008). Flotillin-dependent clustering of the amyloid precursor protein regulates its endocytosis and amyloidogenic processing in neurons. J. Neurosci. Off. J. Soc. Neurosci..

[46] Hattori C., Asai M., Onishi H., Sasagawa N., Hashimoto Y., Saido T.C., Maruyama K., Mizutani S., Ishiura S. (2006). BACE1 interacts with lipid raft proteins. J. Neurosci. Res..

[47] John B.A., Meister M., Banning A., Tikkanen R. (2014). Flotillins bind to the dileucine sorting motif of beta-site amyloid precursor protein-cleaving enzyme 1 and influence its endosomal sorting. FEBS J..

[48] Kamagata E., Kudo T., Kimura R., Tanimukai H., Morihara T., Sadik M.G., Kamino K., Takeda M. (2009). Decrease of dynamin 2 levels in late-onset Alzheimer’s disease alters Abeta metabolism. Biochem. Biophys. Res. Commun..

[49] Hung Y.H., Robb E.L., Volitakis I., Ho M., Evin G., Li Q.X., Culvenor J.G., Masters C.L., Cherny R.A., Bush A.I. (2009). Paradoxical condensation of copper with elevated beta-amyloid in lipid rafts under cellular copper deficiency conditions: Implications for Alzheimer disease. J. Biol. Chem..

[50] Bitsikas V., Riento K., Howe J.D., Barry N.P., Nichols B.J. (2014). The role of flotillins in regulating abeta production, investigated using flotillin 1-/-, flotillin 2-/- double knockout mice. PLoS ONE.

[51] Zhang X.L., Zhao N., Xu B., Chen X.H., Li T.J. (2019). Treadmill exercise inhibits amyloid-beta generation in the hippocampus of APP/PS1 transgenic mice by reducing cholesterol-mediated lipid raft formation. Neuroreport.

[52] Zhu H., Luo L., Hu S., Dong K., Li G., Zhang T. (2014). Treating Alzheimer’s disease with Yizhijiannao granules by regulating expression of multiple proteins in temporal lobe. Neural Regen. Res..

[53] Thomas R.S., Lelos M.J., Good M.A., Kidd E.J. (2011). Clathrin-mediated endocytic proteins are upregulated in the cortex of the Tg2576 mouse model of Alzheimer’s disease-like amyloid pathology. Biochem. Biophys. Res. Commun..

[54] Kong G.K., Miles L.A., Crespi G.A., Morton C.J., Ng H.L., Barnham K.J., McKinstry W.J., Cappai R., Parker M.W. (2008). Copper binding to the Alzheimer’s disease amyloid precursor protein. Eur. Biophys. J..

[55] Guenette S., Strecker P., Kins S. (2017). APP Protein Family Signaling at the Synapse: Insights from Intracellular APP-Binding Proteins. Front. Mol. Neurosci..

[56] Aidaralieva N.J., Kamino K., Kimura R., Yamamoto M., Morihara T., Kazui H., Hashimoto R., Tanaka T., Kudo T., Kida T. (2008). Dynamin 2 gene is a novel susceptibility gene for late-onset Alzheimer disease in non-APOE-epsilon4 carriers. J. Hum. Genet..

[57] Lee S., Mankhong S., Kang J.H. (2019). Extracellular Vesicle as a Source of Alzheimer’s Biomarkers: Opportunities and Challenges. Int. J. Mol. Sci..

[58] Chiasserini D., van Weering J.R., Piersma S.R., Pham T.V., Malekzadeh A., Teunissen C.E., de Wit H., Jimenez C.R. (2014). Proteomic analysis of cerebrospinal fluid extracellular vesicles: A comprehensive dataset. J. Proteom..

[59] Street J.M., Barran P.E., Mackay C.L., Weidt S., Balmforth C., Walsh T.S., Chalmers R.T., Webb D.J., Dear J.W. (2012). Identification and proteomic profiling of exosomes in human cerebrospinal fluid. J. Transl. Med..

[60] Sun B., Dalvi P., Abadjian L., Tang N., Pulliam L. (2017). Blood neuron-derived exosomes as biomarkers of cognitive impairment in HIV. Aids.

[61] Kowal J., Arras G., Colombo M., Jouve M., Morath J.P., Primdal-Bengtson B., Dingli F., Loew D., Tkach M., Thery C. (2016). Proteomic comparison defines novel markers to characterize heterogeneous populations of extracellular vesicle subtypes. Proc. Natl. Acad. Sci. USA.

[62] Kuharic J., Grabusic K., Tokmadzic V.S., Stifter S., Tulic K., Shevchuk O., Lucin P., Sustic A. (2019). Severe Traumatic Brain Injury Induces Early Changes in the Physical Properties and Protein Composition of Intracranial Extracellular Vesicles. J. Neurotrauma.

[63] Stuendl A., Kunadt M., Kruse N., Bartels C., Moebius W., Danzer K.M., Mollenhauer B., Schneider A. (2016). Induction of alpha-synuclein aggregate formation by CSF exosomes from patients with Parkinson’s disease and dementia with Lewy bodies. Brain J. Neurol..

[64] Ou Y.X., Liu F.T., Chen F.Y., Zhu Z.M. (2017). Prognostic value of Flotillin-1 expression in patients with solid tumors. Oncotarget.

[65] Kirsch C., Eckert G.P., Mueller W.E. (2003). Statin effects on cholesterol micro-domains in brain plasma membranes. Biochem. Pharmacol..

[66] Dominguez-Prieto M., Velasco A., Tabernero A., Medina J.M. (2018). Endocytosis and Transcytosis of Amyloid-beta Peptides by Astrocytes: A Possible Mechanism for Amyloid-beta Clearance in Alzheimer’s Disease. J. Alzheimer’s Dis..

[67] Gaudreault S.B., Dea D., Poirier J. (2004). Increased caveolin-1 expression in Alzheimer’s disease brain. Neurobiol. Aging.

[68] Cong L., Kong X., Wang J., Du J., Xu Z., Xu Y., Zhao Q. (2018). Association between SORL1 polymorphisms and the risk of Alzheimer’s disease. J. Integr. Neurosci..

[69] Reitz C. (2013). Dyslipidemia and the risk of Alzheimer’s disease. Curr. Atheroscler. Rep..

